# Supplementary material for: Modified Atkins diet induces subacute selective ragged‐red‐fiber lysis in mitochondrial myopathy patients
Source: EMBO Mol Med. 2016 Sep 19;8(11):1234–47. doi: 10.15252/emmm.201606592 (PMC5090657; doi:10.15252/emmm.201606592)
Supplement: Supplementary file 1 — Expanded View Figures PDF [file EMMM-8-1234-s001.pdf]

Expanded View Figures

Figure EV1. Description of diet compositions, effects of mAD for serum parameters, enzyme histochemistry, and mtDNA amount and mutations.

A Isocaloric mAD composition in PEO patients and control subjects. The amounts of fat, carbohydrates, and proteins are represented as kcal% from the total daily energy expenditure. Values shown as mean and SEM.

B Plasma FGF-21 levels in individual patients and in grouped controls (n = 10) shown as mean ± SD.

C–H Plasma levels of alanine aminotransferase (P-ALT), urea, triglycerides, cholesterol, glucose and insulin in PEO patients and control subjects. Controls (n = 10) are shown as mean ± SD. Dashed line: upper limit of control range.

I COX-SDH histochemical activity analysis on frozen sections from *quadriceps femoris* muscle of the PEO patients in normal diet (ND) and after modified Atkins diet (mAD). Scale bar, 150 μm.

J Skeletal muscle, relative mtDNA amount compared to nuclear single-copy APP gene.

K mtDNA deletion load as a percentage from total mtDNA in PEO muscle.

Data information: Abbreviations: mo, month; yr, year; fP, fasted plasma; COX, cytochrome c oxidase; SDH, succinate dehydrogenase.

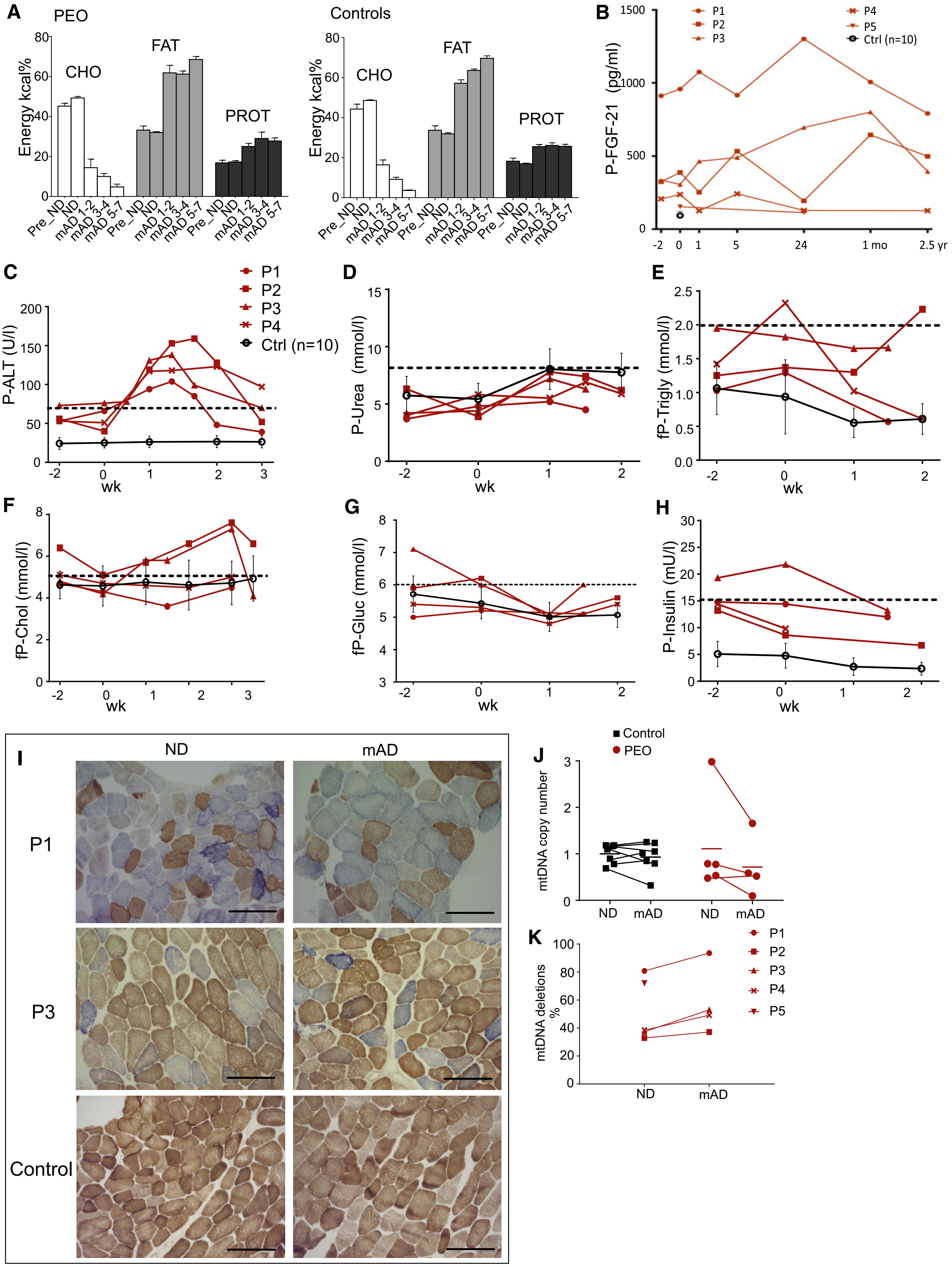

Figure EV1.

 © 2016 The Authors

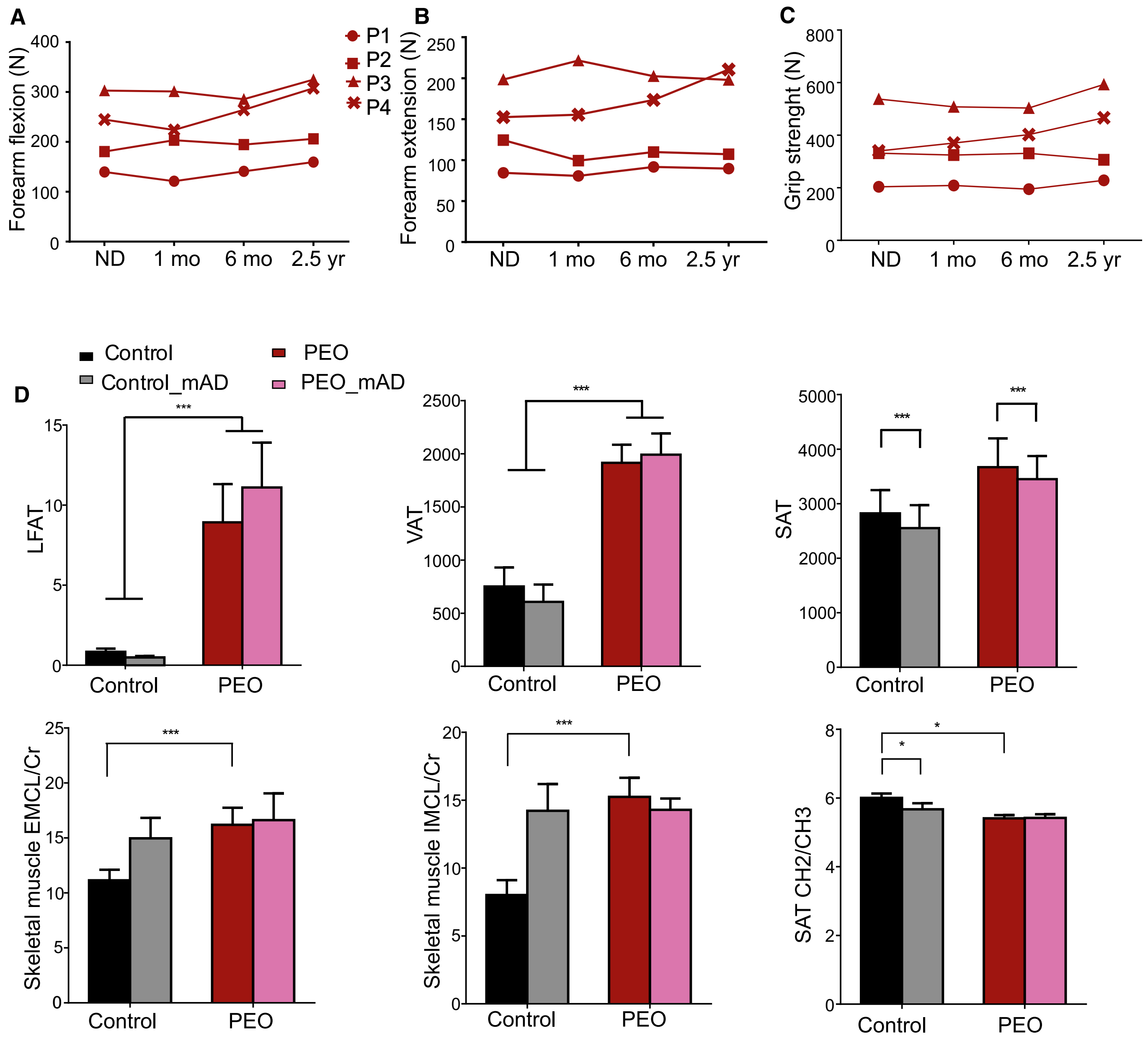

Figure EV2. Muscle strength, *in vivo* lipid contents before and after mAD.

A–C Muscle strength and function of PEO patients on normal diet (ND) as well as 1 and 6 months and 2.5 years after initiation of mAD. P1-4, patients 1–4.

D Magnetic resonance imaging and spectroscopy of liver fat (LFAT), visceral (VAT), and subcutaneous (SAT) adipose tissue, and skeletal muscle of controls and PEO patients on normal diet (ND) and 4 weeks after initiating mAD. EMCL/Cr: extramyocellular lipids (EMCL) or intramyocellular lipids (IMCL) correlated to muscle creatine (Cr). SAT showed decrease of CH2/CH3 ratio, indicating decrease in fatty acid length after the diet in controls. Values shown as mean and SEM. Statistical tests: LFAT, VAT and SAT two-way ANOVA; ECML, IMCL and SAT CH2/CH3 Student's *t*-test (two-tailed). *$P < 0.05$, ***$P < 0.001$.

© 2016 The Authors

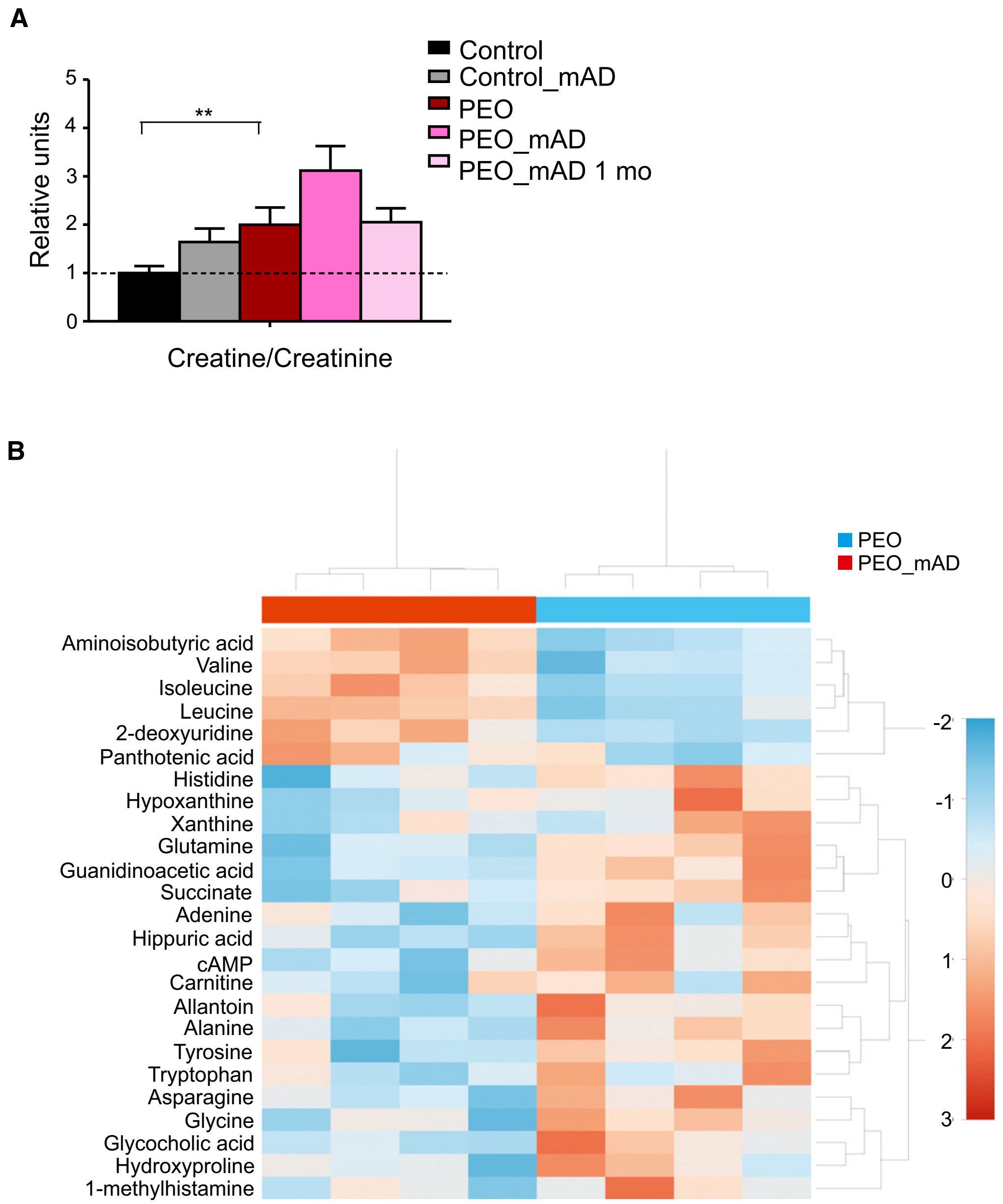

Figure EV3. Plasma metabolomics of PEO patients and controls before and after modified Atkins Diet (PEO_mAD).

A Plasma creatine to creatinine ratio; plasma metabolomics analysis. Values shown as mean and SEM. Student's *t*-test (two-tailed). $^{**}P < 0.01$.

B Heatmap of the 25 most significantly changed metabolites in PEO plasma before and after mAD. Values represent relative to mean in the given data set. Color key: *Z*-score. Hierarchical clustering: vertical axis, metabolites; horizontal axis, patients. Abbreviations: PEO, progressive external ophthalmoplegia; mAD, modified Atkins diet; mo, month after diet initiation.

 © 2016 The Authors
