# Supplementary figures and images for: Modified Atkins diet induces subacute selective ragged‐red‐fiber lysis in mitochondrial myopathy patients
Source: EMBO Mol Med. 2016 Sep 19;8(11):1234–47. doi: 10.15252/emmm.201606592 (PMC5090657; doi:10.15252/emmm.201606592)

Source data for Figure 2C

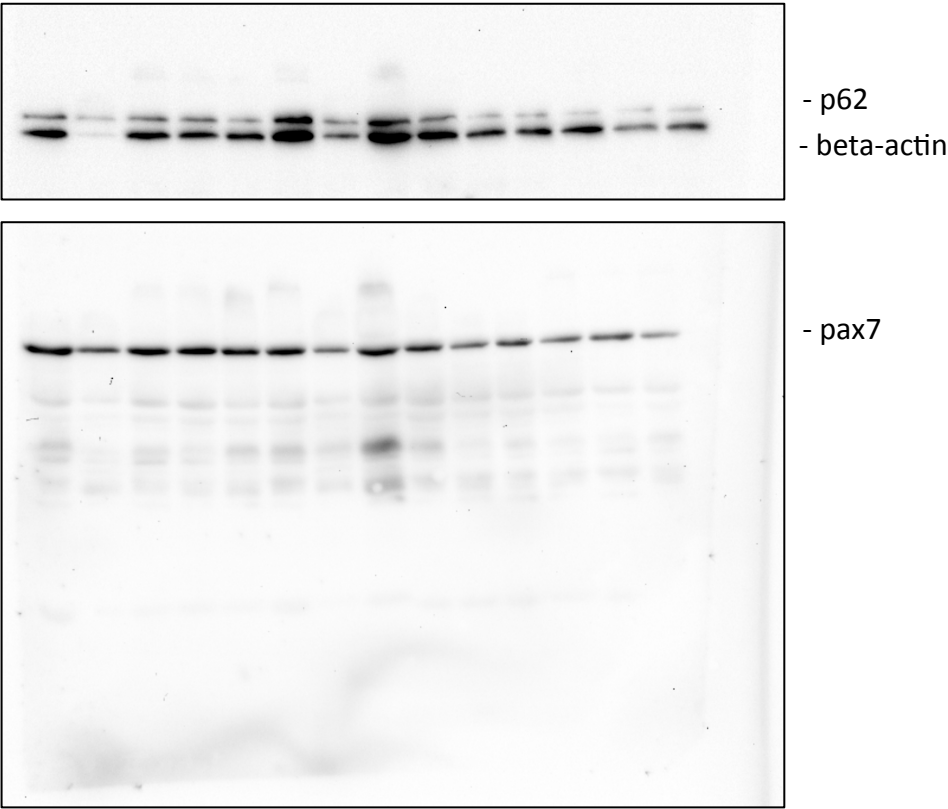

Supplement: Supplementary file 4 — Source Data for Figure 2 [file EMMM-8-1234-s003.pdf]
